# Reply to Lassaunière: On the functional characterization of the Y453F RBD variant found in cluster 5 SARS-CoV-2

**DOI:** 10.1016/j.jbc.2021.101241

**Published:** 2021-10-29

**Authors:** Rafael Bayarri-Olmos, Laust Bruun Johnsen, Anne Rosbjerg, Charlotte Helgstrand, Theresa Bak-Thomsen, Peter Garred, Mikkel-Ole Skjoedt

**Affiliations:** 1 Rigshospitalet, University Hospital of Copenhagen, Copenhagen, Denmark; 2 Novo Nordisk A/S, Måløv, Denmark; 3 Institute of Immunology and Microbiology, University of Copenhagen, Copenhagen, Denmark

We appreciate the opportunity to comment on the letter from Dr Lassaunière.

Lassaunière remarks that the Y453F residue change is not the entire cluster 5 variant, which is in complete agreement with the statements in our article. Only the Y453F residue change is located in the RBD; the other mutations are situated outside and doubtfully impacting the ACE-2 interaction. Throughout our publication (1), we have made it clear that we characterize the impact of the cluster-5 RBD variant (*i.e.*, Y453F). As such, we do not agree with the “*mislabeling…out of context comparison*” stated by Lassaunière.

Because neutralizing antibodies preferentially localize to the RBD (2) and the high degree of correlation between our neutralization ELISA and the plaque reduction neutralization test (PRNT) (3), we conclude that the cluster-5 Y453F-RBD has a minor evasive advantage.

Lassaunière highlights that a cluster-5 report (4) uses a microneutralization assay instead of PRNT. Both are “bona-fide” viral neutralization tests, differing only in their readout (protein N *versus* plaque formation). The assay miswording does not change our conclusions.

Lassaunière claims that our affinity determination is not novel. In a thorough report, Starr *et al*. (5) use surface yeast display for RBD variant expression and flow cytometry to evaluate binding to dimeric ACE-2. The measured affinity (given as “apparent KD” Δlog_10_(K_D, app_)) likely includes the avidity effect, which the authors state themselves. We report the association and dissociation constants describing the 1:1 molecular interaction and believe that our study provides important additional information about the biophysical interaction between Y453F-RBD and ACE2.
